# Lymph Node Staging with Choline PET/CT in Patients with Prostate Cancer: A Review

**DOI:** 10.5402/2011/219064

**Published:** 2011-12-18

**Authors:** Andrea Skanjeti, Ettore Pelosi

**Affiliations:** ^1^SCDU Medicina Nucleare 2, ASO S. Giovanni Battista, Corso Bramante, 88, 10126 Torino, Italy; ^2^Centro PET, IRMET SpA, Via Onorato Vigliani, 89/A, 10138 Torino, Italy

## Abstract

Due to its prevalence, prostate cancer represents a serious health problem. The treatment, when required, may be local in case of limited disease, locoregional if lymph nodes are involved, and systemic when distant metastases are present. In order to choose the best treatment regimen, an accurate disease staging is mandatory. However, the accuracy of conventional imaging modalities in detecting lymph node and bone metastases is low. In the last decade, molecular imaging, particularly, choline PET-CT has been evaluated in this setting. Choline PET represents the more accurate exam to stage high-risk prostate cancer, and it is useful in staging patients with biochemical relapse, in particular when PSA kinetics is high and/or PSA levels are more than 2 pg/ml. The present paper reports results of available papers on these issues, with particular attention to lymph node staging.

## 1. Introduction

Due to its prevalence, prostate cancer represents a medical and social problem [1]. In developed countries, among the male cancer, it is the most common one and the second cause of cancer death in men over 50 years [2, 3]. Thanks to the Prostate Specific Antigen (PSA) screening, in most of the cases, the disease extension at diagnosis is limited. Despite, the relapse is quite common [4], and due to this reason, a strict monitoring of the PSA levels is generally performed [5]. Increases of PSA are the expression of disease relapse, while PSA velocity [3] or PSA doubling time [6] shows a good correlation with the amount of the disease. In presence of relapse, an accurate disease staging is mandatory due to the fact that local and distant relapse present different prognosis and therapeutic approaches. In case of localized or locoregional disease, the salvage radical prostatectomy is indicated (eventually lymphadenectomy), whereas in case of distant metastases, androgen deprivation therapy (ADT) or surveillance is recommended (localized radiotherapy can be of help in those cases with painful bone metastases [4, 7]). 

For several decades, the contribution of nuclear medicine in prostate cancer has been limited to the bone scan, in order to detect bone metastases [8, 9] and to the prostascint (capromab pendetide, a murine monoclonal antibody) in order to detect and potentially treat primary prostate cancer [8]. In the last two decades, PET scan became available and was used in these patients. However, 18F-FDG (the most extensively used tracer in PET imaging) has low sensitivity in identifying prostate cancer, and its physiological urinary excretion reduces specificity in the staging of locoregional disease [8]. Acetate has been studied by few authors in cases of biologic relapse of prostate cancer [8] but even if results seem to be interesting, the little number of studied patients does not let as draw any definitive conclusions. Thanks to its elevated values of sensitivity and specificity, nowadays, the most extensively tracer used in patients with prostate cancer is choline labeled with 11C or 18F. Hara and DeGrado independently developed chemical ways to label choline, initially with 11C [10, 11] and finally with 18F [12]. Another relevant step was represented by the introduction of PET/CT scanners because of the significant increase on sensitivity and specificity in disease detection compared to PET alone [13]. PET/CT choline shows great potential in the staging of prostate cancer, and interesting results have been published; the aim of this paper is to summarize the results of studies evaluating the accuracy of choline PET/CT in the lymph nodal staging of patients with prostate cancer. We reviewed the literature with the help of the Medline search engine PubMed. The search was performed using keywords such as “choline PET” and “prostate cancer” and was limited to works published in English, with particular attention on lymph node staging. Furthermore, manual searches of references listed in the included papers completed our literature search.

## 2. Why Is Choline Interesting?

In order to facilitate their duplication, prostate cells accumulate choline that is useful for the production of phosphatidylcholine, a cell membrane constituent. This ability is more significant in prostate cancer cells [10]. Therefore, in 1998 Hara et al. [10] evaluated 11C-choline PET in few patients with prostate cancer and compared results with 18F-FDG PET. 11C-choline PET showed higher tumour/background ratio with respect to 18F-FDG and false-positive rate was lower. In 2002, the same group developed the synthesis of 18F-fluoroethylcholine, with longer half-life than 11C-choline (120 versus 20 minutes). This new tracer maintains the properties of the previous one, but it can be used even by PET centers without on-site cyclotron [14]. Since then, several studies evaluating these tracers have been published. Hara and DeGrado showed that prostate cancer cells uptake preference was choline > acetate > FDG in aerobic conditions [15]. The group of DeGrado showed that these preferences were valid both in androgen-dependent and -independent cells [16]. The group of Groningen produced another 18F-labeled choline compound (i.e., deshydroxy-fluorocholine) in order to differentiate between the choline kinase activity into the cell and the choline transport from the intercellular space to the cell [17], and another 18F-labeled compound has been synthesized in order to further increase tumour/background ratio [11]. Choline has been evaluated with success in animal studies in order to predict early response to new cancer treatments [18, 19], and thanks to the animal PET studies, the basic research in this field is very active. 

Speaking about cancer diagnosis, the group of Bologna evaluated the accuracy of 11C-choline PET in identifying tumours foci in the prostate compared with sextant biopsy. Positive predictive value was interesting but false-negative rates were too high [20]. However, when evaluating only nodules greater than 5 mm, the sensitivity was good (83%) [21]. The authors described similar specificities between choline PET/CT and MRI spectroscopy [22]. The group of Ulm showed that 11C-choline PET/CT detected and correctly located major areas of prostate cancer with an AUC (area under curve) of 0.89 ± 0.01 for a Standardized Uptake Value (SUV) threshold of 2.65 [23, 24]. However, they underlined that choline PET/CT was not accurate in order to perform a nerve sparing radical prostatectomy due to the low spatial resolution [25]. The group of Hawaii detected prostate cancer with a high degree of confidence (AUC = 0.93) using dynamic 18F choline PET [26, 27], in particular using the (delayed-early) SUVmax. Other authors observed that SUVmax was not able to distinguish with a high degree of confidence between prostate cancer and benign prostate hyperplasia (BPH); in this case, the ratio between SUVmax of prostate cancer and that of pelvic muscle can be useful [28]. The group of Zurich published discouraging results in staging prostate cancer with choline PET/CT, while they showed its usefulness in the staging of patients with radical prostatectomy and PSA levels >2 ng/mL [29].

Picchio and coworkers, from Milan, compared 11C-choline with FDG for restaging prostate cancer [30]. They showed the superiority of choline in respect to conventional imaging and promoted the integration of PET to the conventional diagnostic workup. In another study, they showed that choline could be useful to monitor the response to the antiandrogenic therapy [31]. Evaluating the role of 11C-choline in patients with biochemical relapse, the group of Bologna pointed out that PSA levels (best cutoff 2.43 ng/mL) and PSA kinetics (velocity and/or doubling time) are correlated with the detection rate [32]. Moreover, when used to stage patients with single equivocal bone lesion, PET choline frequently detected further lesions, leading to the modification of the treatment choice [33]. Interestingly, in the restaging of patients with or without biochemical progression, the group of Ulm showed a good accuracy of choline PET (37 out of 49 patients were correctly classified, accuracy: 75.6%) [34]. On the other hand, our group, studied 56 patients with biochemical relapse and found an increase of 18F-choline sensitivity with the increment of PSA levels. We were able to detect recurrences in 24 patients: 4 local recurrences and 20 regional and/or systemic diseases [1].

Other groups studied the role of choline PET/CT in the detection of bone metastases [35, 36] and showed that choline was able to distinguish between viable and sclerotic lesions that present the same aspect on CT scan. Few works evaluated the role of choline PET in radiotreatment planning with promising results [37–39]. Finally, it seems that choline PET could fulfill RECIST criteria to measure tumour response [40]. 

Concluding, given the spatial resolution of PET systems (about 5 mm), choline PET could be useful to detect lesions greater than 5 mm within prostate gland, especially when the acquisition is dynamic, or when early and delayed images are both acquired (dual phase). In patients radically treated, PSA remains the first step in the followup, and only on its basis choline PET can be required. In case of bone metastases choline PET is useful due to its ability to distinguish between viable and sclerotic lesions. Furthermore, choline PET is more sensitive than bone scintigraphy. Finally, PET abilities can be exploited to optimize radiotherapy plan and to evaluate treatment response according to RECIST criteria.

## 3. Choline PET in Lymph Node Staging?

The prostate lymphatics drain into the obturator/internal iliac, external iliac, presacral/perirectal nodes, and, less frequently, into the common iliac nodes [41]. Morphological imaging shows very low sensitivity in this setting [1, 41]; functional/molecular imaging, and so choline PET/CT has been widely investigated in order to define its accuracy.

## 4. Lymph Node Staging in Prostate Cancer

Recently, the group of Linz presented the results of a prospective study with histopathologic confirmation on 130 intermediate and high-risk patients with newly diagnosed prostate cancer studied with choline PET [42]. They reported a per lesion sensitivity of 45% that grows to 66% for lymph node greater than 5 mm; specificity was 96%. By acquiring delayed images, they observed an early wash out of choline in false positive lymph nodes. So the authors concluded that PET is an useful tool to stage patients, especially those with high-risk prostate cancer. The groups of Milan and Bologna studied together the accuracy on lymph node staging of 57 patients with prostate cancer and compared it with clinical nomograms. PET sensitivity, specificity, positive predictive value (PPV), negative predictive value (NPV), and accuracy in the patient-based analysis were 60%, 98%, 90%, 87%, and 87%, while in the lesion-based analysis were 41%, 99.8%, 94%, 97%, and 97%, respectively. The specificity of PET scan was better than the specificity of clinical nomogram; the sensitivity was similar, and even in this study results influenced by the diameter of the lesions [43]. A Danish study [44] prospectively evaluated the accuracy of 18F-choline PET/CT in the detection of lymph node metastases in 25 patients with newly diagnosed intermediate or high-risk prostate cancer. The results were compared with histopathology after lymphadenectomy. PET/CT was positive in four, and three among them were true positive. The authors did not find false-negative results nor observe significant differences between SUVmax in the early and in the late images. They concluded that choline PET was promising, but due to the reduced number of patients, further studies are necessary. Another study reported an accuracy of 93% in the lymph node staging of 67 patients with newly diagnosed prostate cancer [45]. In this latter, choline PET was highly specific (96%) and presented a good sensitivity (80%). On the other hand, discouraging results have been reported by other authors. Steuber et al. [46] evaluated prospectively 18F fluoroethylcholine PET/CT in 20 high-risk patients with prostate cancer prior to the radical prostatectomy and compared PET results with histopathology. They did not observe pathological uptake at PET scan while on histopathology, 31 metastatic lymph nodes were observed. Häcker et al. [47] staged 20 patients with intermediate or high-risk prostate cancer. In 10 cases at least one metastatic lymph node was observed, and 18F-choline PET/CT showed a sensitivity of 10%, a specificity of 80%, and an accuracy of 45%. Budiharto et al. [48], recently, staged 36 patients by the use of 11C-choline PET and compared the results with histopathology. This group found a sensitivity of PET scan under 10% on a per lesion basis and a specificity of 99.7%. Another similar work was performed by Scher et al. [49] that studied 58 patients with the clinical suspicion of prostate cancer. Authors found lymph node metastases in 5 of these cases, and PET scan missed one with micrometastases. The limit of this study is that histopathologic confirmation was available only for positive PET exams and not for the negative ones.

## 5. Lymph Node Detection in Prostate Cancer Relapse

The group of Milan studied 358 patients, and 161 of them showed at least one abnormal choline uptake [2]. Lymph nodes pathological uptake was observed in 107 both in pelvic area (more frequently), and in retroperitoneal area (less frequently) and no significant differences were observed in the accuracy of this exam in patients with ADT compared with those without, whereas the increase of PSA levels correlated with sensitivity in the detection of lymph node metastases. In another study, the same group evidenced an overall PET accuracy of 77% on a per-lesion basis, with sensitivity, specificity, PPV, and NPV of 64%, 90%, 86%, and 72%, respectively (the low NPV was attributed to the limited spatial resolution of the tomograph). Finally in a subsequent study [6], the authors observed that PSA doubling time shorter than 6 months was independently correlated with greater percentage of patients with pathological lymph nodes uptakes. The group of Bologna studied 102 patients in the same setting and found 12 lymph nodes pathological uptakes in 9 patients, all true positive [3]. In this study, the PSA doubling time independently predicted choline PET positivity. Recently, a Dutch group evaluated choline PET in restaging 70 patients with biochemical relapse. They observed pathological uptake in 10 lymph nodes, and among them, only one was false positive [50]. The group of Munich [51] used choline PET/CT to study patients with biochemical relapse of prostate cancer. Thirty-five of 63 patients showed pathological uptakes in choline PET/CT, and among them, 15 demonstrated focal uptake in abdominopelvic lymph nodes. Furthermore, this group compared low-dose CT with diagnostic CT scan, concluding that this latter increased sensitivity and specificity of the PET exam [7]. Rinnab et al. [52] studied 50 patients with biochemical relapse and observed lymph node uptake in 13 of them, however histopathology confirmed only 9 metastases with a predictive positive value of 69% (9/13). Even in this study, the increment of PSA level was related with an increment of PET sensitivity. Schilling et al. [53] studied the positive predictive value of choline PET/CT in 10 patients with biochemical failure and positive lymph nodes at 11C-choline PET scan. Histopathologic results showed metastatic disease in 7 of them (PPV of 70%), and PSA was significantly greater in that group with respect to the group of false positives. Finally, Cimitan et al. [54], using 18F choline, staged 100 patients with biochemical relapse. They found pathological uptake in 54 cases, and in 16 of them, metastatic lymph nodes were observed. Even in this study, choline PET increased its sensitivity in the group of patients with PSA greater than 4 ng/mL.

## 6. Conclusion

To date, there are no concordant results on the role of choline PET in lymph node staging of patients with new diagnosis of prostate cancer. The low PET spatial resolution could be, the most relevant limit. However, it should be pointed out that in this setting, other imaging modalities are less accurate, and so PET represents the best non invasive choice [42, 43]. Taking into consideration the utility in the early detection of bone metastases, we think that choline PET could have a role in this setting, in particular in high-risk patients. Finally, the evaluation of novel treatments has potential advantages from the use of choline PET.

The other setting in which choline PET can have a strategic role is in the staging of patients with biochemical relapse. In this field, the majority of the studies missed an extended histopathology confirmation, and the only value most frequently considered is the PPV. The added value of choline PET could be observed by restaging these patients, in particular those with greater PSA values and higher PSA kinetics. Anyway, well-structured studies with histopathology confirmation may be useful to determine the real accuracy of this exam.
